# Amplifying and Reversing the Chiral Bias in Asymmetric Photo‐Polymerization Reaction

**DOI:** 10.1002/advs.202411439

**Published:** 2024-12-12

**Authors:** Dingdong Liu, Xiangxiang Xu, Zeyu Feng, Chutian Zhang, Jialei Li, Yifan Xie, Jingguo Li, Hongli Zhang, Gang Zou

**Affiliations:** ^1^ Key Laboratory of Precision and Intelligent Chemistry School of Chemistry and Materials Science University of Science and Technology of China Hefei Anhui 230026 China; ^2^ CAS Key Laboratory of Urban Pollutant Conversion Department of Environmental Science and Engineering University of Science and Technology of China Hefei Anhui 230026 China

**Keywords:** asymmetric photo‐polymerization, circularly polarized light, chiral metal nanoparticles, chirality synergy and competition, polydiacetylene

## Abstract

Circularly polarized light (CPL) is inherently chiral and is regarded as one possible source for the origin of homochirality. Coincidentally, chiral metal nanoparticles have great prospects in asymmetric photochemical reactions since they can enhance the chiral light‐matter interactions. Nonetheless, little is known about how the spin angular momentum of light competes with the chiral electromagnetic field in the vicinity of a chiral nanoparticle during the chiral induction and amplification process. Here, an asymmetric photo‐polymerization system is presented where the chiral bias can be selectively regulated by the combination of chiral fields CPL and Ag nanoparticles, either constructively or destructively. Synergistic chiral amplification effect is observed when the handedness of both are matched, while the opposite‐handed CPL can overrule the polymer helicity during the chain propagation process when the chiral near‐field is weak. There is a bifurcation point in the chiral bias for the orthogonal control of the polymer helicity during the asymmetric photo‐polymerization process, favoring for programmable chiroptical micropatterns and multi‐channel independent information encoding. This work not only highlights opportunities for selectively regulating the asymmetric photopolymerization but also is highly valuable for fundamental understanding of the symmetry breaking in photochemical reactions.

## Graphical abstract

The symmetry breaking photo‐polymerization reaction can be selectively regulated by the chiral information inherited from the CPVL or chiral AgNPs, either constructively or destructively, which served as “far‐field photonic sergeant” and “near‐field sergeant” respectively.

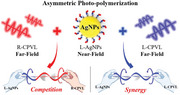

## 1 Introduction

**Scheme 1:**
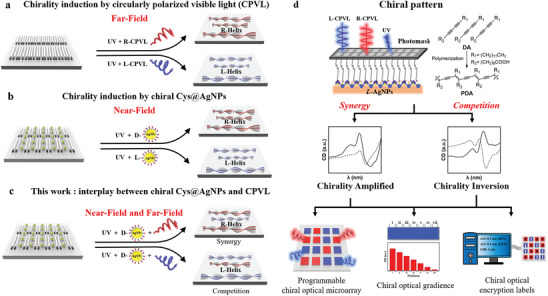
Schematic representation of strategies for asymmetric photosynthesis of chiral materials, a) conventional CPVL only, b) chiral Gaps only, and c) the newly proposed strategy, coupling of CPVL and chiral AgNPs. d) The synergy strategy integrates the power of both ‘far‐field photonic sergeant’ and ‘near‐field sergeant’, which enables a higher degree of programmability of the polymeric chiral patterns and features wide applications in various fields.

Symmetry breaking, leading to a specific helical conformation (left or right handedness) of biological structure, plays a vital role in many unique biology functions.^[^ ^1^, ^2^ ^]^ Inspired by biological helices, the precise synthesis of chiral organic molecules,^[^ ^3^ ^]^ inorganic nanomaterials,^[^ ^4^ ^]^ helical supramolecular assemblies^[^ ^5^ ^]^ and polymers^[^ ^6^, ^7^ ^]^ have attracted enormous attentions due to their special properties and various potential applications in biosensing, enantioselective catalysis,^[^ ^8^ ^]^ chiroptical metamaterials and so on.^[^ ^9^, ^10^ ^]^ In the past decades, many strategies have been developed to synthesize chiral materials based on conventional chemistry‐driven chirality transfer using chiral reactant,^[^ ^11^ ^]^ chiral catalyst,^[^ ^12^ ^]^ chiral solvent,^[^ ^13^ ^]^ or by applying external physical driving force, such as stirring,^[^ ^14^ ^]^ vortex motion,^[^ ^15^ ^]^ or circularly polarized light (CPL).^[^ ^16^ ^]^ Thereinto, CPL is inherently chiral and has been regarded as possible origins for the evolution of homochirality in nature.^[^ ^17^ ^]^ CPL has been widely utilized as the chiral source in asymmetric synthesis of chiral organic molecules,^[^ ^18^, ^19^ ^]^ regulation the chiral morphology of inorganic nanomaterials,^[^ ^20^ ^]^ and helical structure modulation in polymers^[^ ^21^, ^22^ ^]^ or supramolecular systems.^[^ ^23^, ^24^ ^]^ We reported that the asymmetric polymerization reactions of DA monomer could be realized by using circularly polarized visible light (CPVL) as a “far‐field photonic sergeant”,^[^ ^25^ ^]^ which could effectively control the helical structure of polydiacetylene (PDA) chain during the chain propagating process (**Scheme** **1**a). However, in most cases, only limited enantiomeric excess were obtained due to the small Kuhn anisotropy factors.^[^ ^26^ ^]^ In addition, the photo‐to‐matter chiral transfer was assumed to be another chiral inducer in the asymmetric photopolymerization process. Compared with the well‐established technique using CPL, the effect of chiral induction based on the chiral electromagnetic field in the vicinity of chiral nanoparticles is not well understood as the enhanced optical asymmetry is usually restricted in a very narrow region near chiral metal nanoparticles. Chiroptical responses can be greatly enhanced, owing to the local chiral electromagnetic fields generated by optical excitation of chiral plasmonic nanoparticles.^[^ ^27^, ^28^ ^]^ Recently, we have demonstrated that chiral metal nanoparticles as the “near‐field sergeant” could impart chiral bias and directly dictate the screw sense of polymers via asymmetric polymerization (Scheme 1b).^[^ ^29^ ^]^ Consequently, it would be highly desirable to selectively regulate the chiral bias in asymmetric photo‐polymerization via the arbitrarily coupled CPL with chiral metal nanoparticles.

Studies in this field are mainly about the enantioselective synthesis via a single chiral source, yet, it remains underexplored on how a system responds when two chiral inputs are simultaneously transmitted. Recently, Meijer et al. reported the competition between chiral solvents and chiral monomers in the helical bias of supramolecular polymers.^[^ ^30^ ^]^ Seo et al. reported that CPL could override and amplify asymmetry in supramolecular helices.^[^ ^31^ ^]^ In our previous work,^[^ ^25^ ^]^ we also demonstrated asymmetric photopolymerization could be mediated by two different types of CPL, circularly polarized ultraviolet light (CPUV) and circularly polarized visible light. However, very little is known about the coupling effect by the combination of CPVL and chiral metal nanoparticles on the overall chirality, either constructively or destructively.

Herein, we reveal that the chiral electromagnetic fields generated upon UV excitation of chiral Ag nanoparticles could act as the “near‐field sergeant” by inducing symmetry breaking during the photoinitiation process, and further compete/cooperate with the CPVL (“far‐field photonic sergeant”) in chain propagation process. Matching the handedness direction of CPVL to those of chiral electromagnetic fields could amplify polymer chirality cooperatively, while a mismatch offset their contribution or even overrule the helical preference of the polymeric chain (Scheme 1c). Specifically, when the UV irradiation is above certain threshold intensity, the polymer helicity persists, even in the presence of the opposite handed CPVL. While when the generated chiral field is weak, CPVL could even inverse the polymer helicity during the chain propagation process and thereby overrule the helical preference that is inducted by the chiral metal nanoparticles at the first place. Therefore, the programmable chiral PDA patterns with tailorable chiroptical response and circularly polarized luminescence distributions could be achieved, which provides unique platform in the fields of chiroptical devices, chiral information encoding, and anti‐counterfeiting materials (Scheme 1d).This concept will inspire the orthogonal control of chiral bias in asymmetric photopolymerization and will be of great fundamental importance for a deeper understanding of the mirror symmetry breaking in asymmetric photochemical reaction.

## 2 Results and Discussion

**Figure 1:**
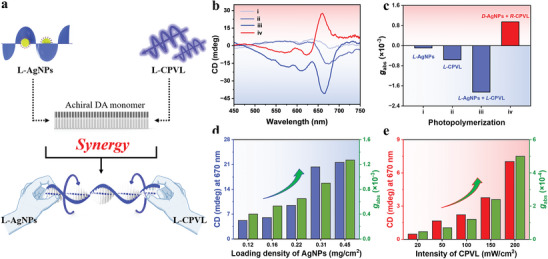
a) Schematic of synergy strategy by simultaneously utilizing chiral AgNPs and CPVL with the same handedness. b) CD characterizations of PDA films by utilizing: i) *L*‐AgNPs; ii) *L*‐CPVL; iii) the combination of *L*‐CPVL and *L*‐AgNPs, and iv) *R*‐CPVL and *D*‐AgNPs as the symmetry breaker in asymmetric polymerization process, and c) their respective *g*_abs_. d) CD intensities at 670 nm by varying the distribution density of *D*‐AgNPs. The *R*‐CPVL intensity was maintained at 100 mW cm^−2^. e) CD intensities at 670 nm by varying the intensity of *R*‐CPVL. The distribution density of *D*‐AgNPs solution was maintained at 0.45 mg cm^−2^. The intensity of UV light was 10 µW cm^−2^ in all above cases. The volume of AgNPs solution was 200 µL.

To achieve asymmetric photopolymerization reactions, either CPVL only, chiral AgNPs only or the combination of both were employed as the symmetry breaker. Chiral AgNPs (≈20 nm in size, Figure S1, Supporting Information) were synthesized in analogy to the previous procedure,^[^ ^32^ ^]^ and exhibited expected CD signals (positive and negative Cotton effect appeared at 256 and 291 nm) at the corresponding inter‐band absorption position (Figure S2, Supporting Information). After drop‐casting of these chiral AgNPs on the quartz substrate surface, a thick film of the DA monomer was subsequently deposited using vacuum deposition method (see method section for more details), and the thickness of DA monomer is ≈5 µm by SEM characterization (Figure S3, Supporting Information). Thin film X‐ray diffraction (XRD) characterizations indicated that the monomer film is highly crystalline (Figure S4, Supporting Information) where the DA molecules are packed in an ordered lamellar fashion. Such a molecular‐level closed packing is favorable for their subsequent polymerization to form PDA. As mentioned above, upon UV excitation of chiral AgNPs, local chiral electromagnetic fields will be generated which can impart chiral bias during the chain initiation stage and eventually trigger the synthesis of helical PDA. Meanwhile, CPVL itself can also promote the helical chain formation. It is anticipated that the enantioselectivity in asymmetric photo‐polymerization might be further amplified by utilizing chiral AgNPs and the CPVL with same handedness as the symmetry breaker simultaneously, as outlined in **Figure** **1**a. The asymmetric photopolymerization reaction was initialed either by CPVL, UV (excitation of chiral AgNPs) or the combination of both, experimental apparatus is shown in Figure S5 (Supporting Information). After photopolymerization, all films turned blue and exhibited intense absorption maximum at 595 and 652 nm, confirming the formation of PDA chains (Figure S6, Supporting Information). When these films were subjected to CD characterizations, obvious CD signals could be observed at the corresponding absorption band for PDA backbone, with a positive and negative Cotton effect appeared at 635 and 673 nm with a crossover at 650 nm (Figure 1b). To exclude possible linear dichroism and linear birefringence effect, CD characterizations were performed by rotating the films (perpendicular to the CD beam) with different angles or by flipping amplification effect during the asymmetric photopolymerization reaction. This synergistic effect has two contributing components. On the one hand, local chiral electromagnetic field will be generated in the close vicinity of *L*‐AgNPs upon UV irradiation them. The variation of the CD signal was marginal at all rotation angles or even upon flipping (Figure S7, Supporting Information), indicating that the recorded CD signals of the films should be ascribed to the formation of helical PDA chains.

As shown in Figure 1b, weak CD signals could be observed for the samples photopolymerized utilizing left‐handed CPVL only (*L*‐CPVL) or UV excited *L*‐type chiral AgNPs (*L*‐AgNPs) as the symmetry breaker. In the presence of combined *L*‐CPVL and UV (excitation of *L*‐AgNPs), the intensity of CD signals increased drastically (Figure 1b, curve iii), indicating the formation of enriched left‐handed helical PDA chains. This means the symmetry breaking photopolymerization reaction can be boosted under synergistic far‐field CPVL and local chiral electromagnetic field in the vicinity of chiral AgNPs. Similarly, by combining of right‐handed CPVL (*R*‐CPVL) and UV excited *D*‐cysteine coated chiral AgNPs (*D*‐AgNPs), opposite and amplified CD signals could be observed, suggesting the preferred formation of right‐handed helical PDA chains (Figure 1b, curve iv).

To further evaluate the dissymmetry amplification effect, we defined the absorption dissymmetry factor (*g*_abs_) as *g*_abs_ = (ɛ_L_‐ɛ_R_)/[(ɛ_L_+ɛ_R_)/2], where |*g*_abs_|<2. The ɛ_L_ and ɛ_R_ were defined as the absorption coefficients of left‐ and right‐handed circularly polarized light, respectively. As shown in Figure 1c, an obvious enhancement in the obtained *g*_abs_ values could be achieved when asymmetric photopolymerization reactions were carried out under simultaneous *L*‐CPVL and UV (excitation of *L*‐AgNPs) illuminations. Compared to the case using only *L*‐CPVL or UV, 3.5‐fold and 10‐fold enhancement in *g*_abs_ values at 670 nm could be achieved, respectively, indicating the existence of synergistic this near‐metal‐surface chiral field is in favor of producing more *L*‐type helical PDA oligomers during the chain initiation stage. On the other hand, this enantioselectivity can be amplified during the chain propagation stage upon simultaneous irradiation with the *L*‐CPVL due to the chiral matching principle.^[^ ^31^ ^]^ To further explore the origin of the synergistic effect, the asymmetric photopolymerization experiments were carried out by either varying the distribution density of chiral AgNPs or modulating the light intensity of CPVL while keeping the rest constant. As shown in Figure 1d, in the case of the combined *R*‐CPVL and UV irradiated *D*‐AgNPs, PDA films exhibited stronger CD signals upon increasing the distribution density of *D*‐AgNPs. At higher *D*‐AgNPs surface loading, more chiral‐active “hot spots” are available in the hybrid films, the collective chiral electromagnetic field nearby these “hot spots” would experience nonlinear cumulative effect, which in turn, will impart stronger chiral bias during the chain initiation stage and induce a greater chirality in the PDA film. Meanwhile, the increasing of the *R*‐CPVL intensity also results in nonlinear chiral enhancement in CD intensities of the final PDA films (Figure 1e), where this far‐field is believed to be effective during the chain propagation process. Therefore, the synergistic chiral amplification effect is realized by the collaboration between the near‐metal‐surface chiral field upon UV irradiation and the far‐field CPVL, where respective helical PDA oligomer formation and helical PDA backbone propagation processes can be facilitated simultaneously.

**Figure 2:**
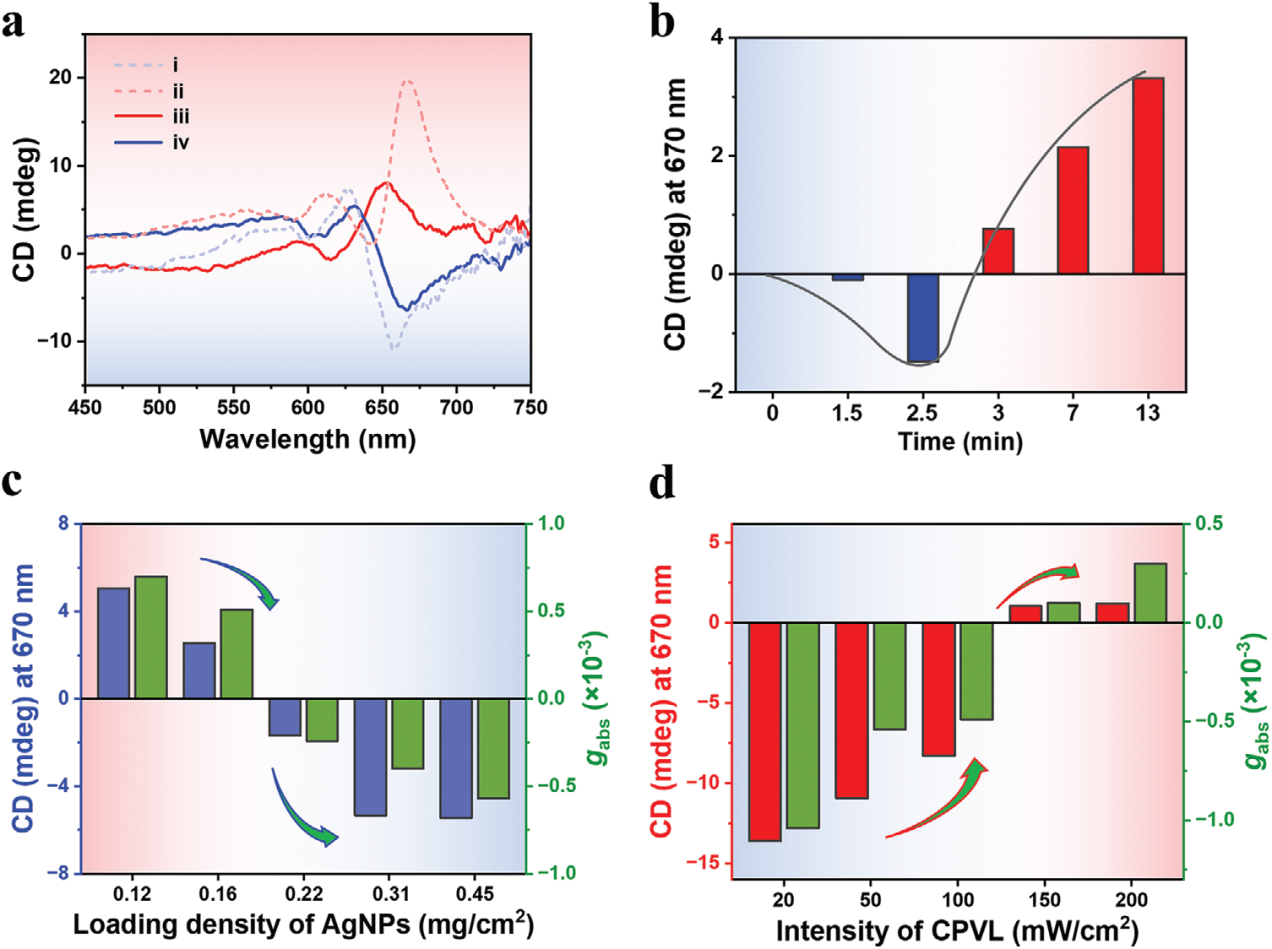
a) CD characterizations of PDA films by utilizing: i) *L*‐AgNPs; ii) *R*‐CPVL; iii) the combination of *R*‐CPVL and higher *L*‐AgNPs loading; iv) the combination of *R*‐CPVL and lower *L*‐AgNPs loading as the symmetry breaker. b) Dynamic CD signal of competition strategy by simultaneously utilizing *L*‐AgNPs and *R*‐CPVL: i) 1.5 min; ii) 2.5 min; iii) 3 min; iv) 7 min; v) 13 min. c) CD intensities at 670 nm by varying the distribution density of *L*‐AgNPs solution. The *R*‐CPVL intensity was 100 mW cm^−2^. d) CD intensities at 670 nm by varying the intensity of *R*‐CPVL. The distribution density of *L*‐AgNPs solution was 0.45 mg cm^−2^. The intensity of UV light was 1 µW cm^−2^ in all above cases.

In addition to the synergistic chiral amplification effect, it is also interesting to explore the possible competitive effects when the asymmetric photopolymerization were carried out by utilizing opposite handed chiral AgNPs and CPVL (**Figure** **2**a; Figure S8, Supporting Information). When either *L*‐AgNPs or *R*‐CPVL was employed as the symmetry breaker, opposite CD signals for PDA backbone was observed as expected (Figure 2a, curve i and ii). While when integrating the opposite handed *R*‐CPVL and *L*‐AgNPs as the chiral sources, the CD signals of the PDA backbone can change their sign depending on the relative strength of the opposite chiral fields (Figure 2a). Specifically, when the chiral field strength of *R*‐CPVL is stronger than that of the collective near‐metal‐surface chiral field, in other word, high *R*‐CPVL intensity and low *L*‐AgNPs loading, the final PDA films will exhibit positive CD signals (Figure 2a, curve iii), indicating the preferred formation of right‐handed helical PDA chains. While, in the case of stronger collective near‐metal‐surface chiral field (high *L*‐AgNPs concentration) and weak chiral field of *R*‐CPVL, opposite CD signals would be obtained (Figure 2a, curve iv), indicating that the preferred formation of left‐handed helical PDA chains. Moreover, in order to probe the progress of the asymmetric photopolymerization reaction while simultaneously applying the opposite handed *R*‐CPVL and *L*‐AgNPs symmetry breakers, time‐resolved UV–vis and CD spectra were recorded. As expected, the absorption peak corresponding to polydiacetylene increased with the illumination time, and its evolution kinetics was depicted in Figure S9 (Supporting Information). In the early stage (up to 1.5 min), the chiral PDA oligomers followed the handedness of the chiral AgNPs, as a weak negative CD signal at ≈670 nm is present (Figure 2b, negative CD signal at 670 nm), indicating the preferred formation of left‐handed helical PDA chains. Interestingly, after the early stage (at ≈2.5 to 3 min), the chirality of the PDA film underwent a sudden transition from left‐handed to right‐handed (Figure 2b, CD signal at 670 nm from negative to positive). This chirality inversion is induced by the R‐CPVL during the chain propagation process. Under such circumstances, upon extended illumination, the right‐handed CD signal continued to grow (Figure 2b). Overall, the final chirality of the PDA film exhibits a tradeoff behavior between the two competing opposite chiral fields. As an example, when the intensity of *R*‐CPVL was fixed to 100 mW cm^−2^, the overall enantioselectivity of the PDA film seems to be dominated by the chiral AgNPs at higher distribution density, and by CPVL when the distribution density of chiral AgNPs was low (Figure 2c). Similarly, when the distribution density of chiral AgNPs was fixed to be 0.45 mg cm^−2^ and UV intensity was 1 µW cm^−2^, the overall enantioselectivity of the PDA film will be overridden by strong CPVL irradiation (Figure 2d). All above results indicated that the overall enantioselectivity of a PDA film is the result of dynamic interplay of the far‐field of CPVL and the near‐field of chiral AgNPs.

Finally, it should be noted here that above certain threshold (chiral AgNPs 0.45 mg cm^−2^, UV 10 µW cm^−2^), helicity of PDA determined by the chiral electromagnetic field nearby chiral AgNPs (“near‐field photonic sergeant”) persisted despite a high CPVL intensity of 200 mW cm^−2^ was applied (Figure S10, Supporting Information), which may be due to the fact that the near‐field effect is too strong and the applied 200 mW cm^−2^ opposite handed CPVL is not strong enough to overrule the formed enantioselectivity.

**Figure 3:**
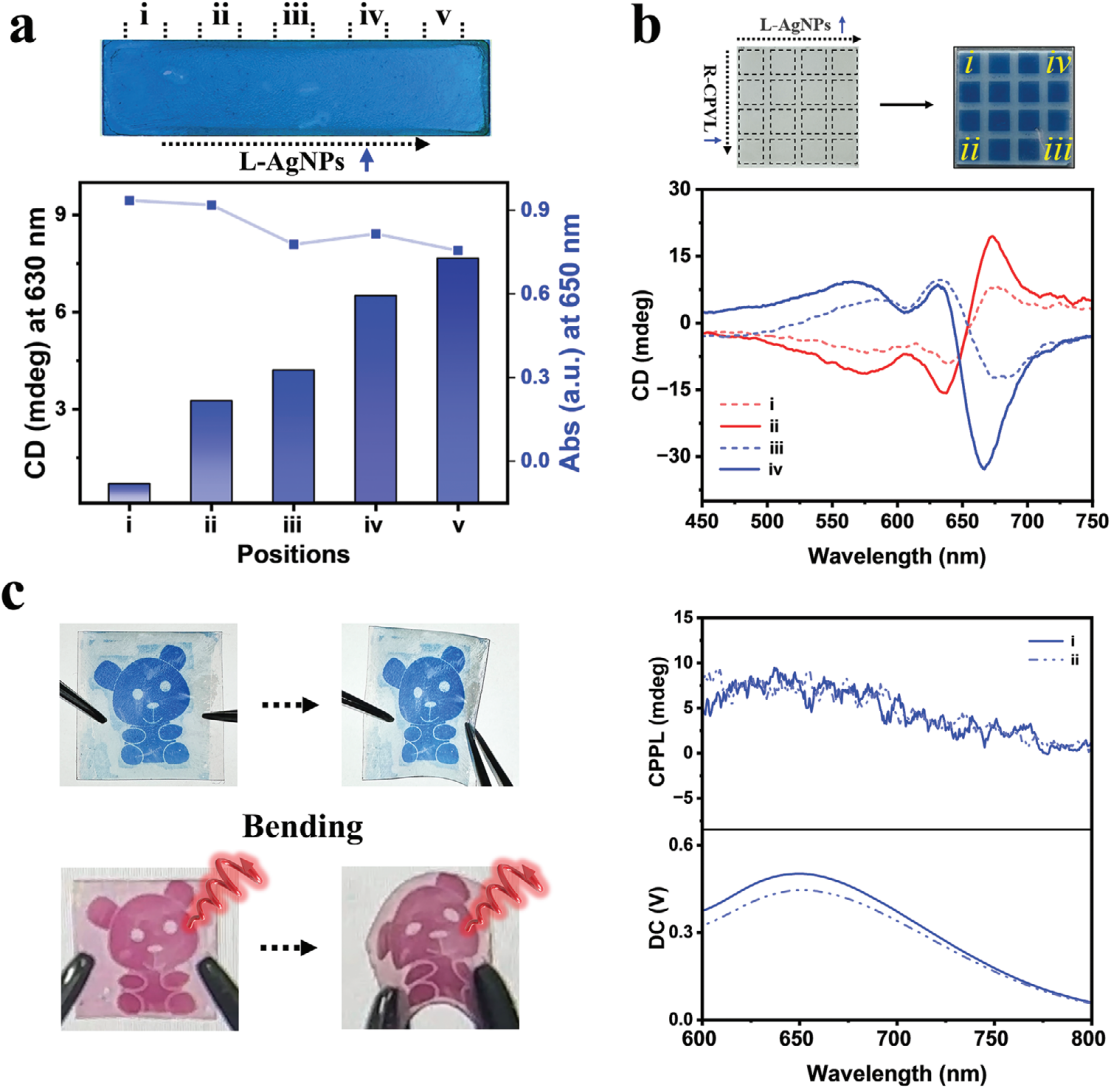
a) Fabricating the continuous gradient of chirality in PDA films. b) A matrix of PDA patches with tailorable chirality by simply varying the relative ratio of “far‐field photonic sergeant” (*R*‐CPVL irradiation) and “near‐field sergeant” (chiral *L*‐AgNPs), and the corresponding CD spectra. c) Programmable flexible chiral PDA patterns in blue and red phase (after thermochromic phase transition), and their circularly polarized photoluminescence performance: i) before and ii) after bending.

As has been demonstrated above, by choosing an appropriate ratio of “far‐field photonic sergeant” (CPVL irradiation) and “near‐field sergeant” (the amount of chiral AgNPs), it is possible to fine‐tune the chiral asymmetry of PDA films through balancing the chiral synergistic and competitive effects. Surfaces with chiral gradients are ubiquitous in nature and have potential applications in biosensing, chiral separations, etc., but it is very challenging to produce those surfaces with traditional lithographic techniques.^[^ ^29^ ^]^ While *L*‐AgNPs could be easily pre‐printed with chirality gradient by inkjet printing techniques. After vacuum deposition of the DA monomer films, such a chirality gradient in the *L*‐AgNPs layer could be transferred to the PDA film upon the irradiation with UV (5 µW cm^−2^) and *L*‐CPVL (100 mW cm^−2^). The resulted PDA film is visually uniform, as also indicated by the comparable absorption at different lateral locations but owns distinct chirality gradient as suggested by the CD signals (**Figure** **3**a). Based on the competitive effects between *R*‐CPVL and the chiral electromagnetic field nearby *L*‐AgNPs, both sign and the intensity of CD signal could be fine‐tuned by simply controlling the ratio between “far‐field photonic sergeant” and “near‐field sergeant”. Using a similar method, a matrix of square patches with gradient loading of *L*‐AgNPs was fabricated. DA monomer films were prepared on the surface of above *L*‐AgNPs matrix, and followed by irradiation with UV (1 µW cm^−2^) and a gradient distributed *R*‐CPVL (from 100–200 mW cm^−2^). A matrix of square patches of PDA with each patch featuring distinct chiroptical property could be obtained, as shown in Figure 3b. Moreover, in situ photopatterning of chiroptical films could be realized by selectively irradiate certain regions of DA monomer films. When utilizing flexible PVA as substrate instead of quartz, this photopatterned chiroptical film can be transferred onto curved surfaces. As shown in Figure 3c, a “bear” symbol of PDA pattern could be written on a flexible PVA film by the aforementioned method through a photomask plate. Interestingly, both absorption and CD signals of the PDA pattern remains constant upon bending of the film, indicating a robust chiroptical performance (Figure S11, Supporting Information). Moreover, upon heating at 80 °C, PDA pattern turned to red during the annealing process (Figure 3c, bottom), owing to the thermochromic phase transition of PDA chains. CD signals of the PDA pattern was maintained but showed a corresponding blue shift of the UV–vis absorption band (Figure S11b, Supporting Information). Interestingly, the red PDA pattern emitted a spatial distribution of circular polarized fluorescence, and the bending hardly affected their circularly polarized photoluminescence (CPPL) performance (Figure 3c; Figure S12, Supporting Information), which might be useful in the fields of 3D display, chiroptical encryption, and so on.

**Figure 4:**
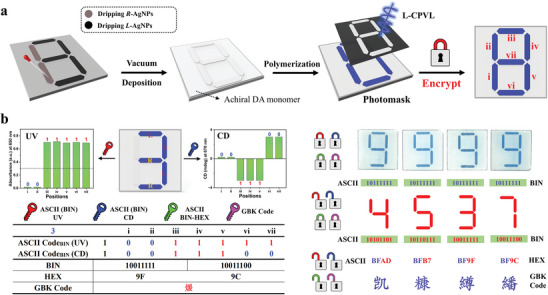
a) Schematic diagram of the fabrication process for the PDA patterned films. b) Schematic of PDA patterned films with independent absorbance and CD signals for the ASCII encoding and the corresponding decryption process.

Recently, multi‐channel independent information encoding has attracted increasing attention due to its ability to significantly enhance the capacity and security of information encoding.^[^ ^33^ ^]^ As mentioned above, by just varying the relative ratio of “far‐field photonic sergeant” (CPVL irradiation) and “near‐field sergeant” (chiral nanoparticles), it is possible to obtain patterns with desired absorbance and chirality distribution independently. Therefore, we further validated the usage of them by performing information encoding in a 2D pattern, as shown in **Figure** **4**. Seven patches of chiral PDA with independent absorbance and CD signal could be achieved, together they can compile a symbol for American Standard Code for Information Interchange (ACSII) as the cryptographic algorithm. Strong absorbance (>0.3) and weak absorbance (<0.3) were defined as “1” and “0” respectively for the apparent encoding, while a positive signal (CD_670nm_ value > 0) and a negative signal (CD_670nm_ value < 0) were defined as “0” and “1” respectively for the covert encoding. Thus, the first layer of encoded information could be read by analyzing both the absorbance and CD signals of the designed area (Figure S13, Supporting Information). Subsequently, binary and hexadecimal ASCII were used for the second and third decryptions of different areas, and the combination of numbers and letters obtained from hexadecimal decoding through the Chinese Internal Code Specification (GBK code) encoding yields the ultimate correct information—Chinese characters. As expected, the encoded information could only be correctly displayed when both the absorption and chirality of the encoding pattern was read out. For instance, when interpreting this combination code, the overt code could be read as “ 3 ” with visual color difference or UV–vis characterization, which is binary ASCII “ 1 0011111 ”, and the covert code “ 7 ” can be obtained through CD characterization, with binary ASCII “1 0011100”. Decrypting both codes into hexadecimal ASCII results in the combinations “ 9F ” and “ 9C ”, which, when cross‐integrated and decrypted through GBK encoding, yield the code for the Chinese character “煖”, achieving multiplex anti‐counterfeiting decryption. Therefore, programmable irradiation using chiral particles as encryption keys can achieve information encoding in a 2D pattern. We anticipate that in the future, color segments will be generated by various stimuli, and thus, existing technology can be extended to multi‐channel independent information encoding when external stimuli also serve as encryption keys for information.

## 3 Conclusion

In summary, we demonstrate that symmetry breaking photo‐polymerization reaction of DA monomers could be selectively regulated by the chiral information inherited from the CPVL or chiral AgNPs, either constructively or destructively. The asymmetric photo‐polymerization occurs via a chain‐initiation and propagation process. The chiral electromagnetic fields that generated upon UV excitation in the close vicinity of chiral Ag nanoparticles can break the symmetry during the formation of left‐ and right‐handed diacetylene dimer and accelerate the formation of helical PDA chain with a specific screw sense that coincides with the handedness of chiral AgNPs. Matching the handedness direction of CPVL to those of chiral electromagnetic fields can further amplify polymer chirality cooperatively, while the opposite handed CPVL can bias the asymmetric photo‐polymerization during the chain propagation process or even overrule the helical preference of final PDA films. Moreover, in situ chiral patterning and multichannel encryption could be successfully achieved, favoring for future applications in 3D display, chiroptical encryption, and chiral optoelectronics. Our findings are not only of great importance for a deep understanding of the symmetry breaking in asymmetric photo‐polymerization reactions, but also open a new pathway for designing programmable chiroptical patterning.

## Conflict of Interest

The authors declare no conflict of interest.

## Supporting information



Supporting Information

## Data Availability

The data that support the findings of this study are available in the supplementary material of this article.
