# Supplementary material for: Amplifying and Reversing the Chiral Bias in Asymmetric Photo‐Polymerization Reaction
Source: Adv Sci (Weinh). 2024 Dec 12;12(5):2411439. doi: 10.1002/advs.202411439 (PMC11792003; doi:10.1002/advs.202411439)
Supplement: Supplementary file 1 — Supporting Information [file ADVS-12-2411439-s001.docx]

Supplementary Information

**Amplifying and Reversing the Chiral Bias in Asymmetric Photo-polymerization Reaction**

Dingdong Liu^[a]^, Xiangxiang Xu^[a]^, Zeyu Feng^[a]^, Chutian Zhang^[a]^, Jialei Li^[a]^, Yifan Xie^[a]^, Jingguo Li*^[b]^, Hongli Zhang*^[a]^ and Gang Zou*^[a]^

**1.Experimental section**

**1.1 Materials**

10,12-Pentacosadiynoic acid (DA), Silver nitrate (AgNO_3_, 99%), *D*/*L*-Cysteine (*D*/*L*-Cys) and ethylene glycol were obtained from Sigma-Aldrich (St. Louis, MO, USA). PVP (Polyvinylpyrrolidone K30) and acetone were obtained from Sinopharm Chemical Reagent Co., Ltd. All other chemicals and solvents and reagents were of analytical grade and used as received. Milli-Q water (18.2MΩ cm) was used in all cases. Silver nanoparticles (AgNPs) were synthesized in analogy to the previous procedure ^[32]^.

**1.2 Measurements**

All UV−Vis absorption spectra were recorded on a Shimadzu UV-2700 PC spectrophotometer. Transmission electron microscopy (TEM) characterizations were recorded on a HITACHI HT-7700. Dynamic light scattering (DLS) experiments were carried out on Brookhaven NanoBrook 90Plus PALS. Powder X-ray diffraction (XRD) was collected on a Japan Rigaku Miniflex 600 rotation-anode X-ray diffractometer equipped with graphite-monochromatized Cu Kα radiation. Circular dichroism (CD) spectra were characterized by using JASCO CD spectrometer J-1500. The *g*_abs_ values at maximum CD signal were calculated to evaluate the formed helical PDA. Circular polarized luminescence characterizations were recorded using a JACSO CPL-300 spectrometer.

**1.3 Asymmetric** **photopolymerization reactions**

In a typical protocol, the Quartz Flake shall be soaked in sulfuric acid for one day, then rinsed with deionized water. Subsequently, hydrophilic substrate was obtained by using Plasma Cleaner. 200 μL of chiral AgNPs solution was drop-casted on a quartz substrate (1.5 cm * 1.5 cm) as the chiral inductor for the asymmetric photo-polymerization reactions. Subsequently, the films of DA monomer with a thickness of about 5 μm were prepared on the chiral AgNPs coated quartz substrates by conventional vacuum deposition method with a deposition rate of 0.01 nm s^-1^. The pressure during the deposition of DA monomers was kept below 1.0 × 10^-1^ Pa. The polymerization experiments were carried out upon the irradiation of unpolarized 254 nm UV light (light intensity: 0 to 10 μW/cm^2^), and left-handed or right-handed circularly polarized visible light (CPVL, 405 nm, 0 to 200 mW/cm^2^). Polymer films with different optical activities were achieved by concentration of chiral AgNPs, the intensity of 254 nm UV light and left- or right-handed CPVL, and the irradiation time.


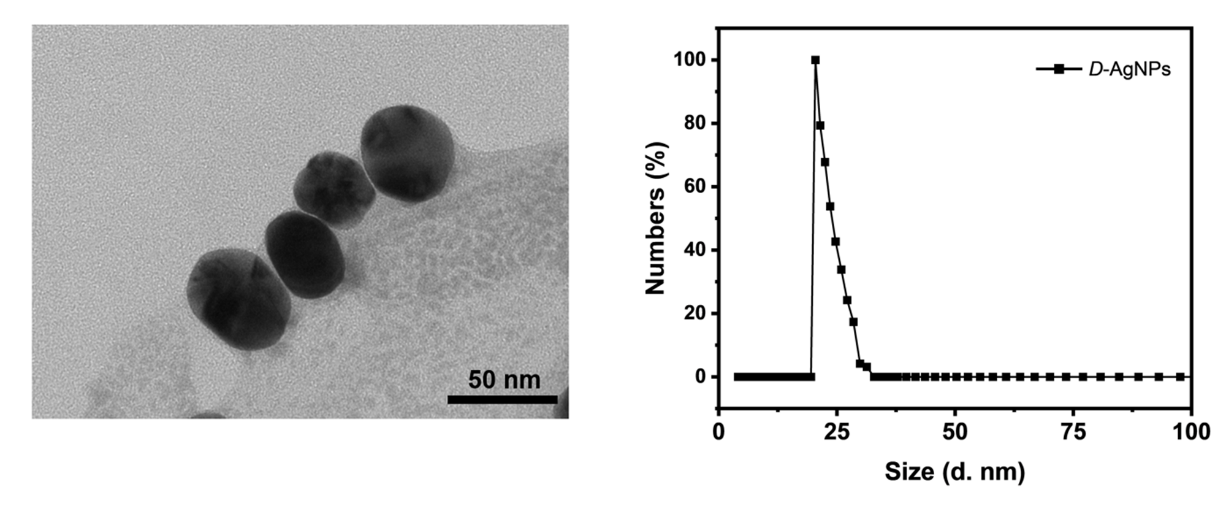


**Figure S1.** TEM and DLS characterization of *D*-AgNPs.


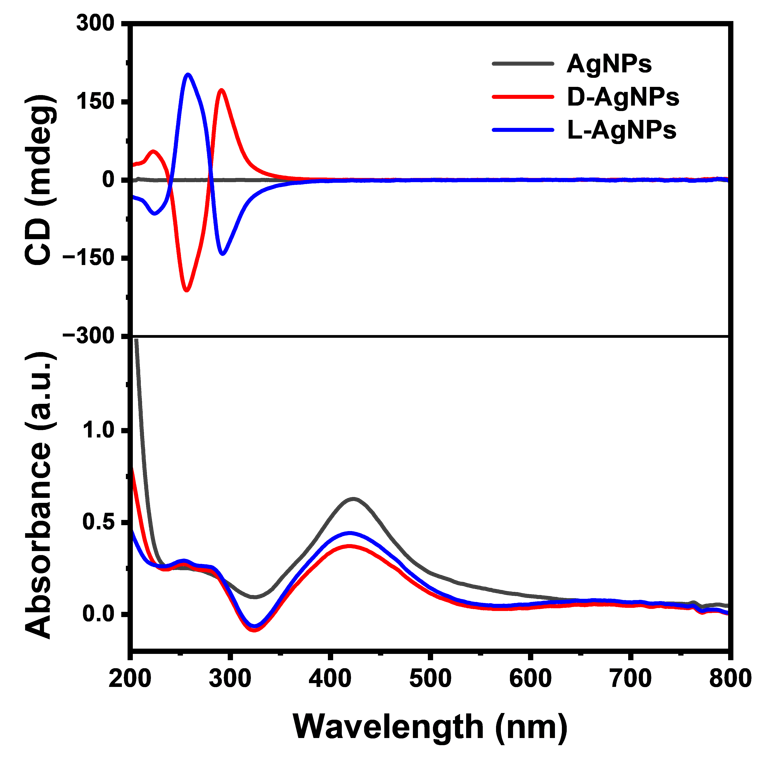


**Figure S2.** CD and UV-Vis spectra of AgNPs, *L*- AgNPs and *D*-AgNPs.





**Figure S3.** SEM characterization of the diacetylene monomer film.


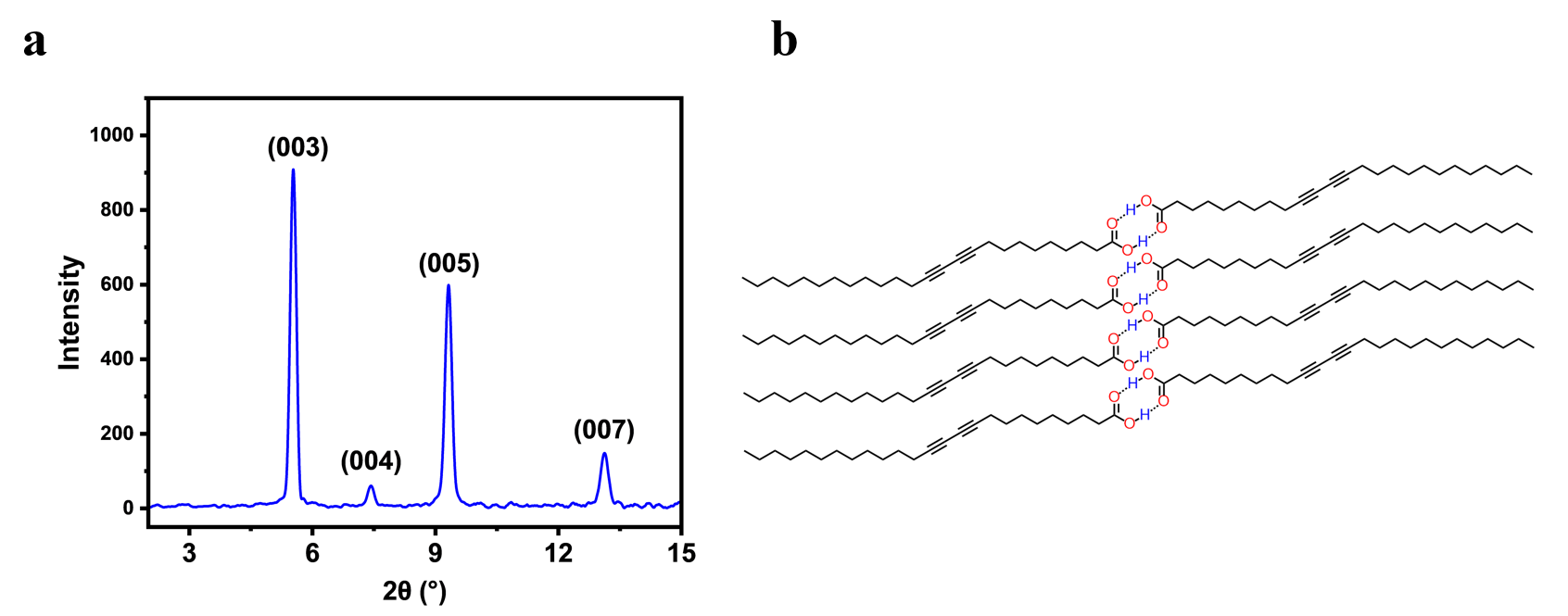


**Figure S4**. (a) XRD characterization of diacetylene monomer film and (b) Structure diagram of diacetylene monomer arrangement.


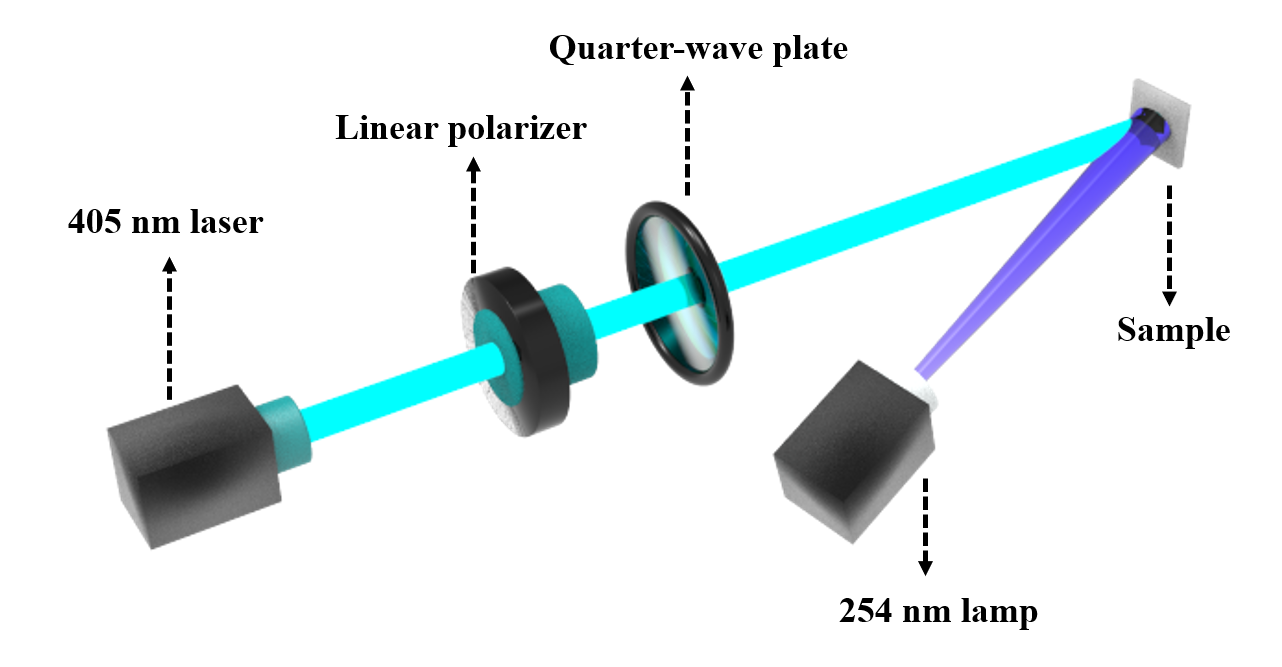


**Figure S5**. Schematic diagram of the experimental optical path of UV and CPVL shining on the sample at the same time.


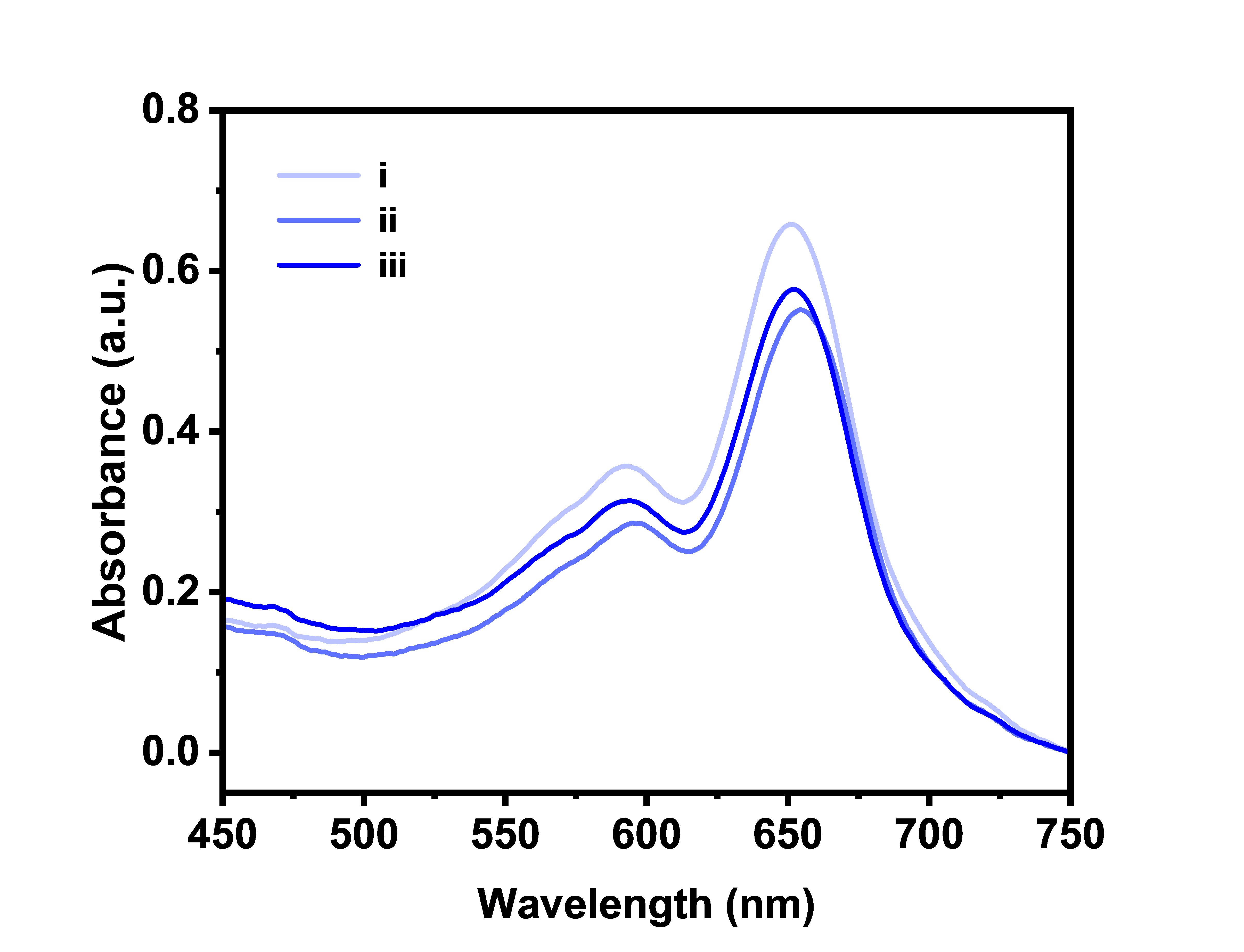


**Figure S6.** UV-Vis spectra of PDA films by utilizing: (i) *L*- AgNPs; (ii) *L*-CPVL and (iii) the combination of *L*-CPVL and *L*-AgNPs.


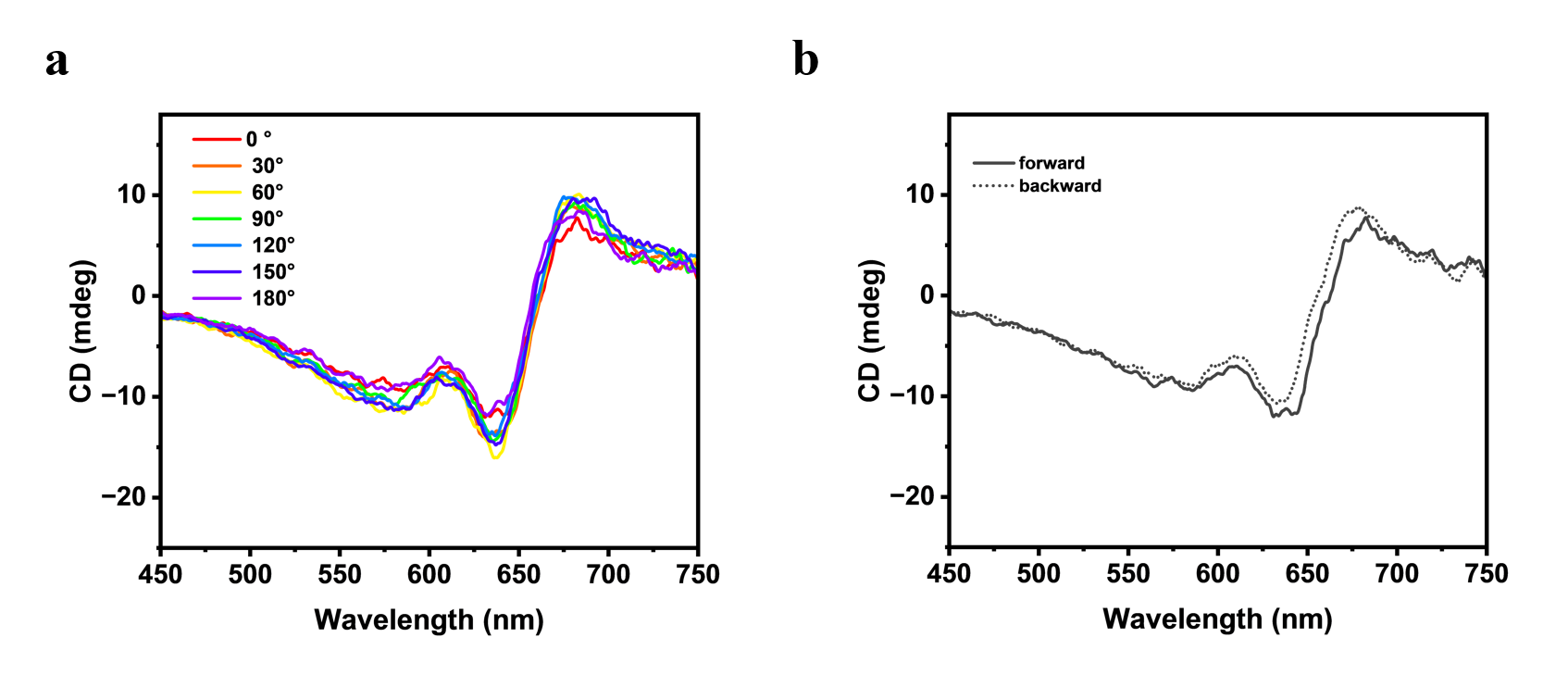


**Figure S7.** (a) CD characterization of a PDA film by utilizing the combination of *L*-CPVL and *L*-AgNPs at various rotation angles from 0 ^o^ to 180^o^. (b) CD spectra of the forward and backward of PDA film.

**
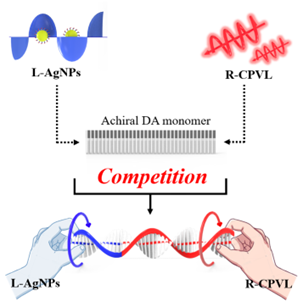
**

**Figure S8.** Schematic of competition strategy by simultaneously utilizing chiral AgNPs and CPVL with the opposite handedness.


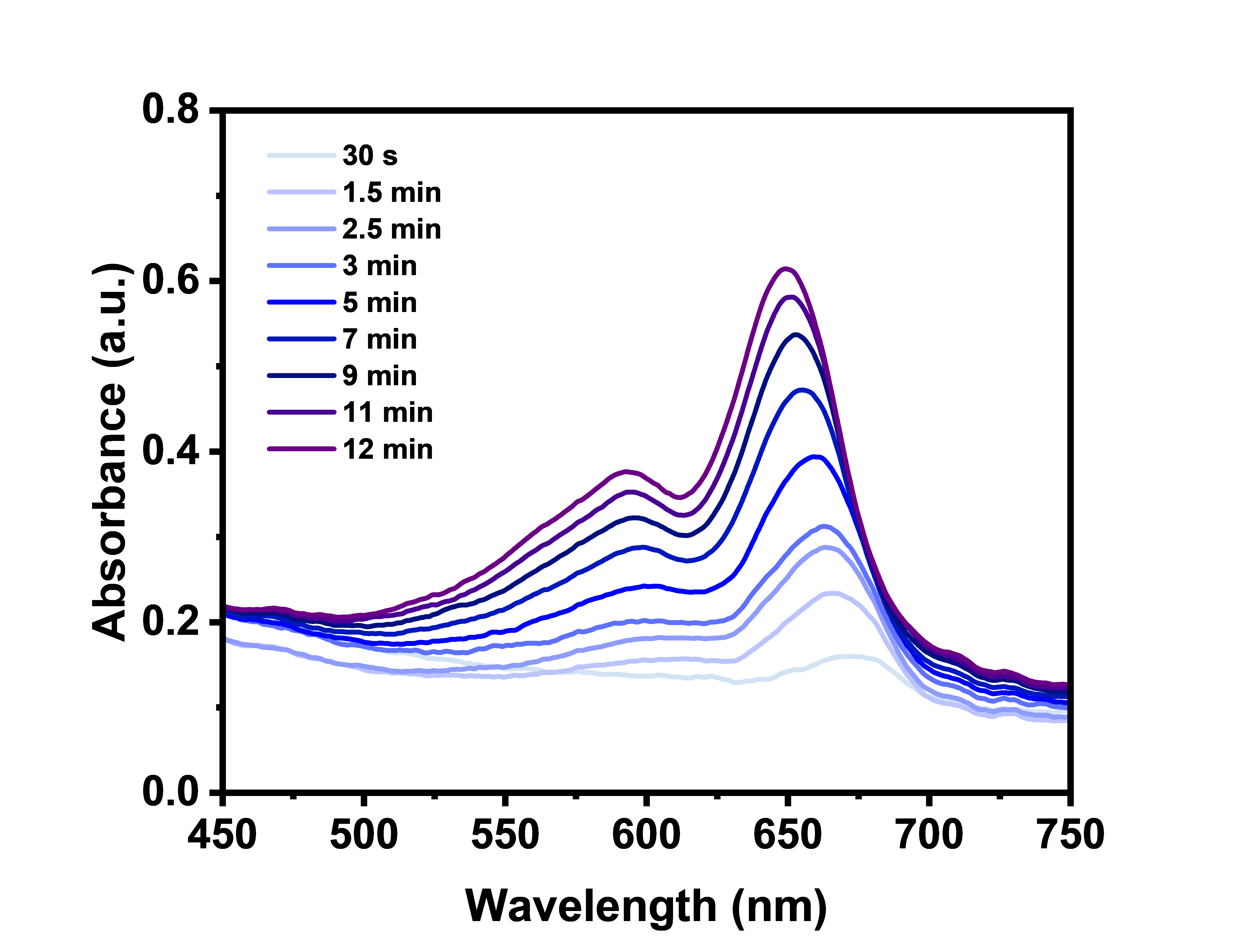


**Figure S9.** UV-Vis signal of competition strategy.

**
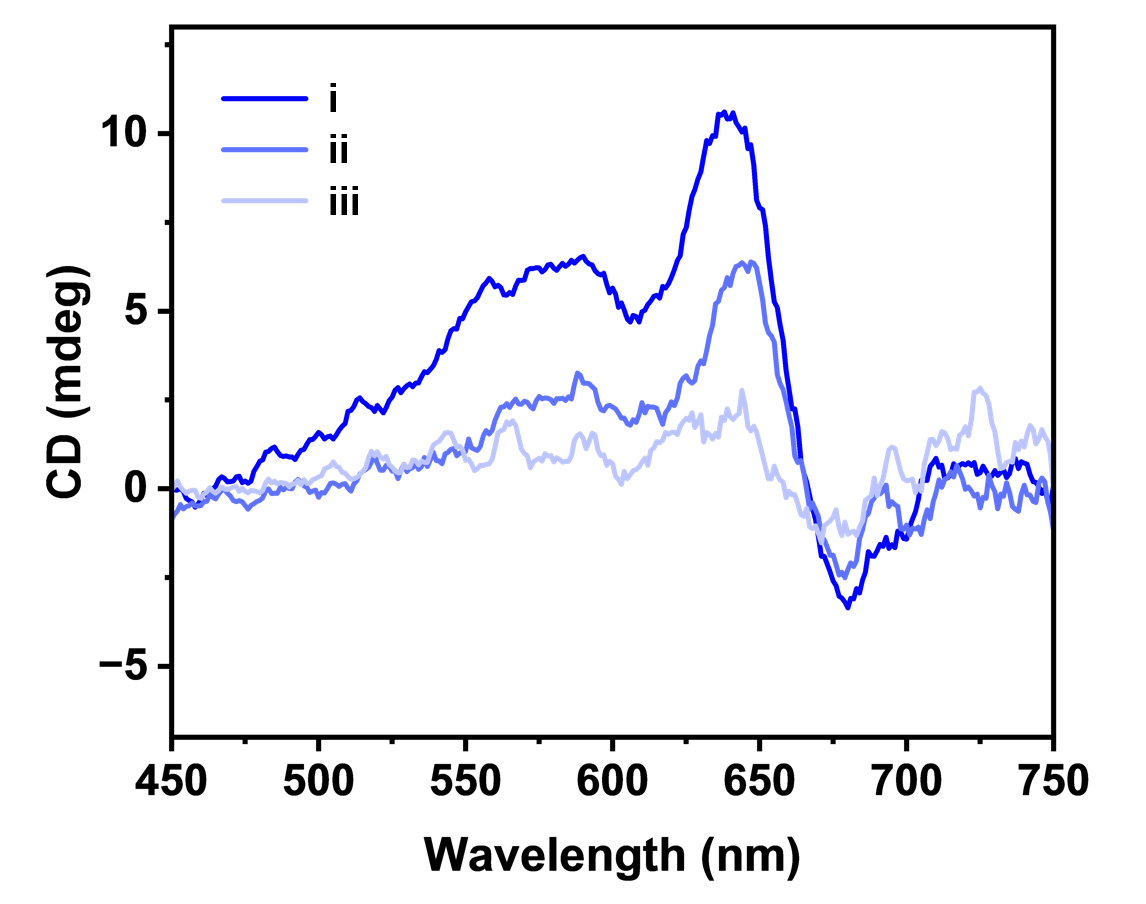
**

**Figure S10.** Threshold of the helicity is determined by (i) *L*-AgNPs, (ii) *L*-AgNPs + *R*-CPVL (100 mW/cm^2^) and (ii) *L*-AgNPs + *R*-CPVL (200 mW/cm^2^).

**
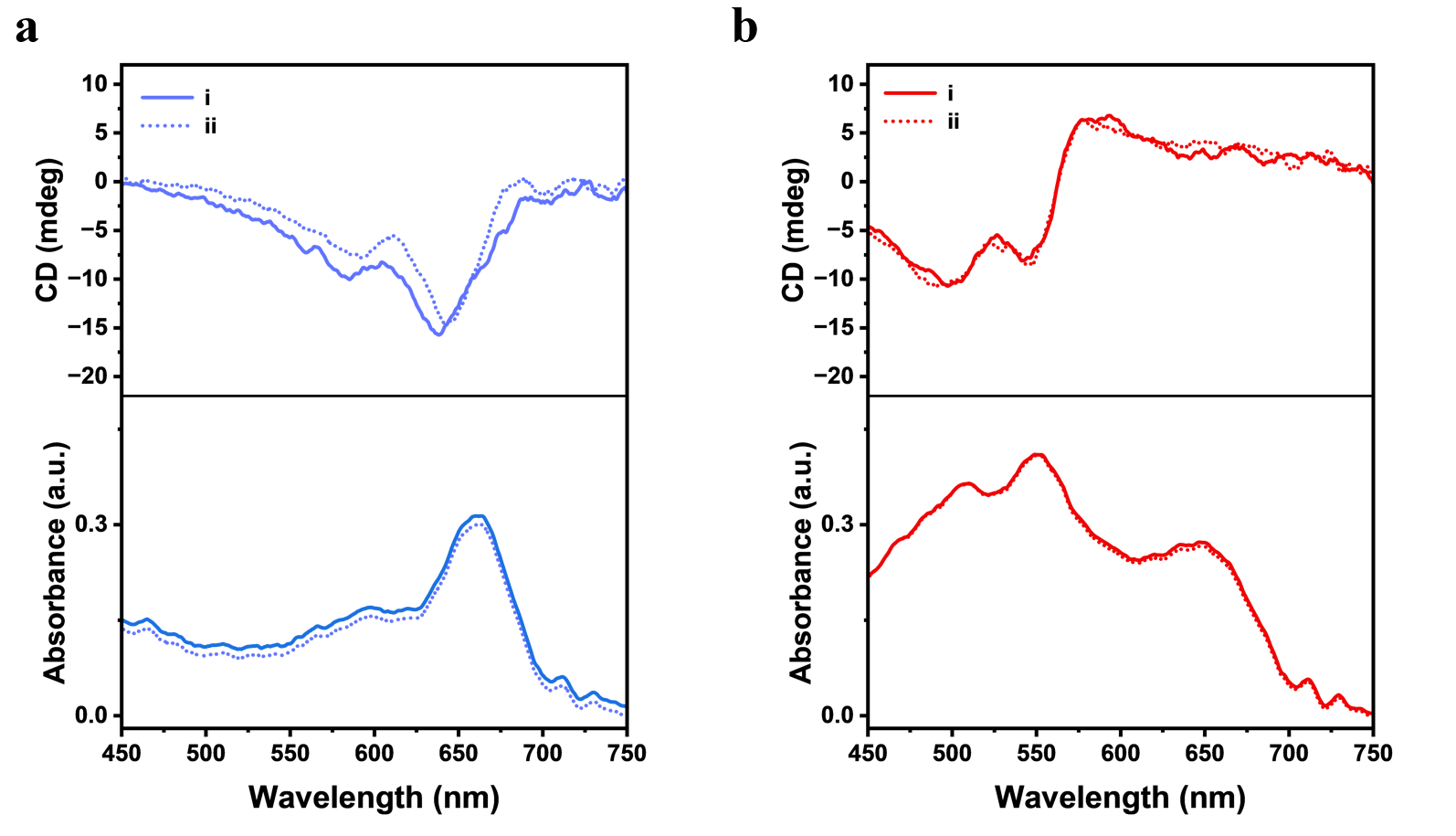
**

**Figure S11.** (a) CD and UV-vis spectra of chiral blue-phase PDA films: (i) before and (ii) after bending. (b) CD and UV-vis spectra of chiral red-phase PDA films: (i) before and (ii) after bending. The blue phase PDA completely transitions to red phase PDA after being heated at 80 °C for 2 min.


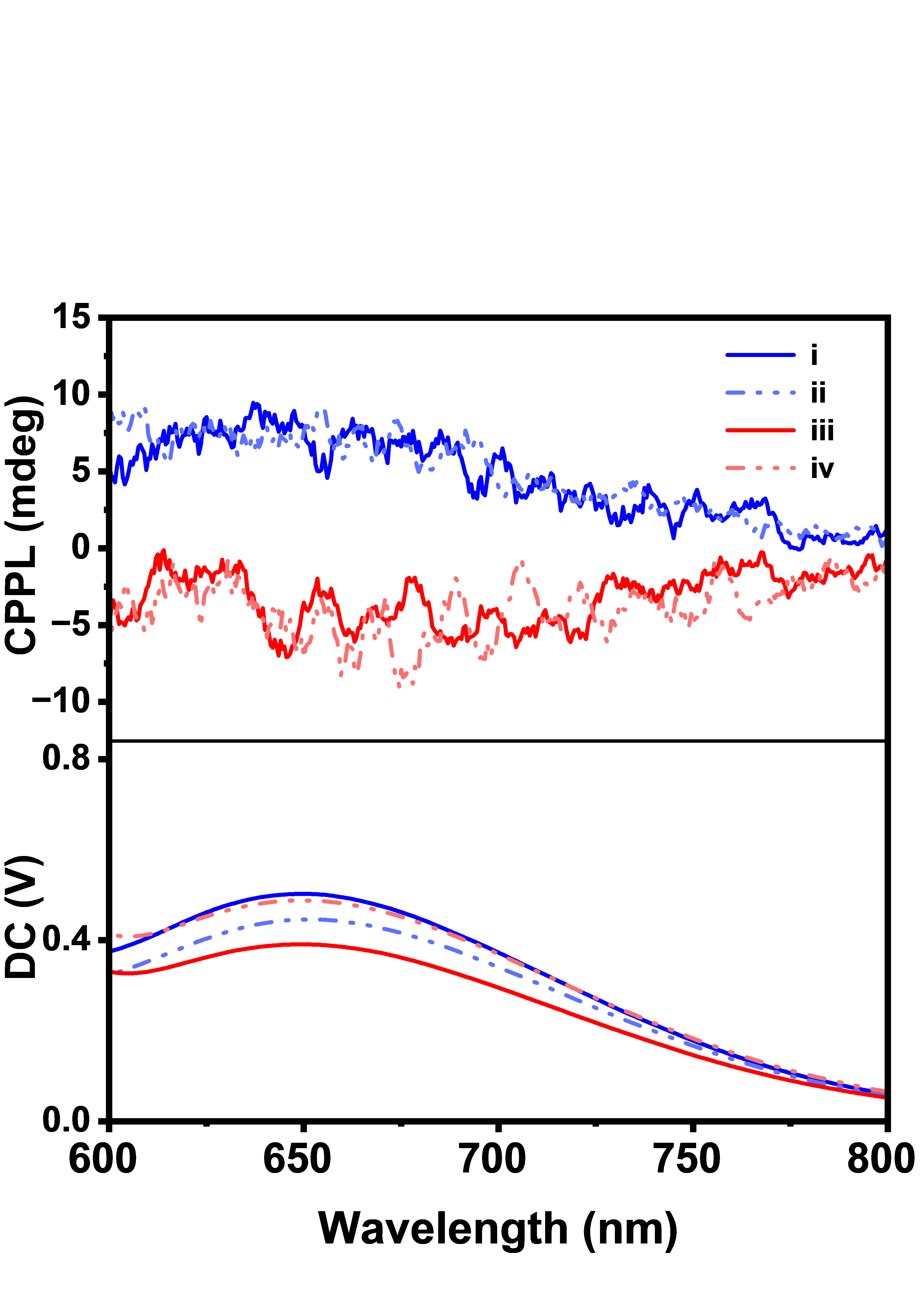


**Figure S12.** The circularly polarized photoluminescence spectra of left-handed red phase PDA films (i) before and (ii) after bending, and right-handed red phase PDA films (iii) before and (iv) after bending.


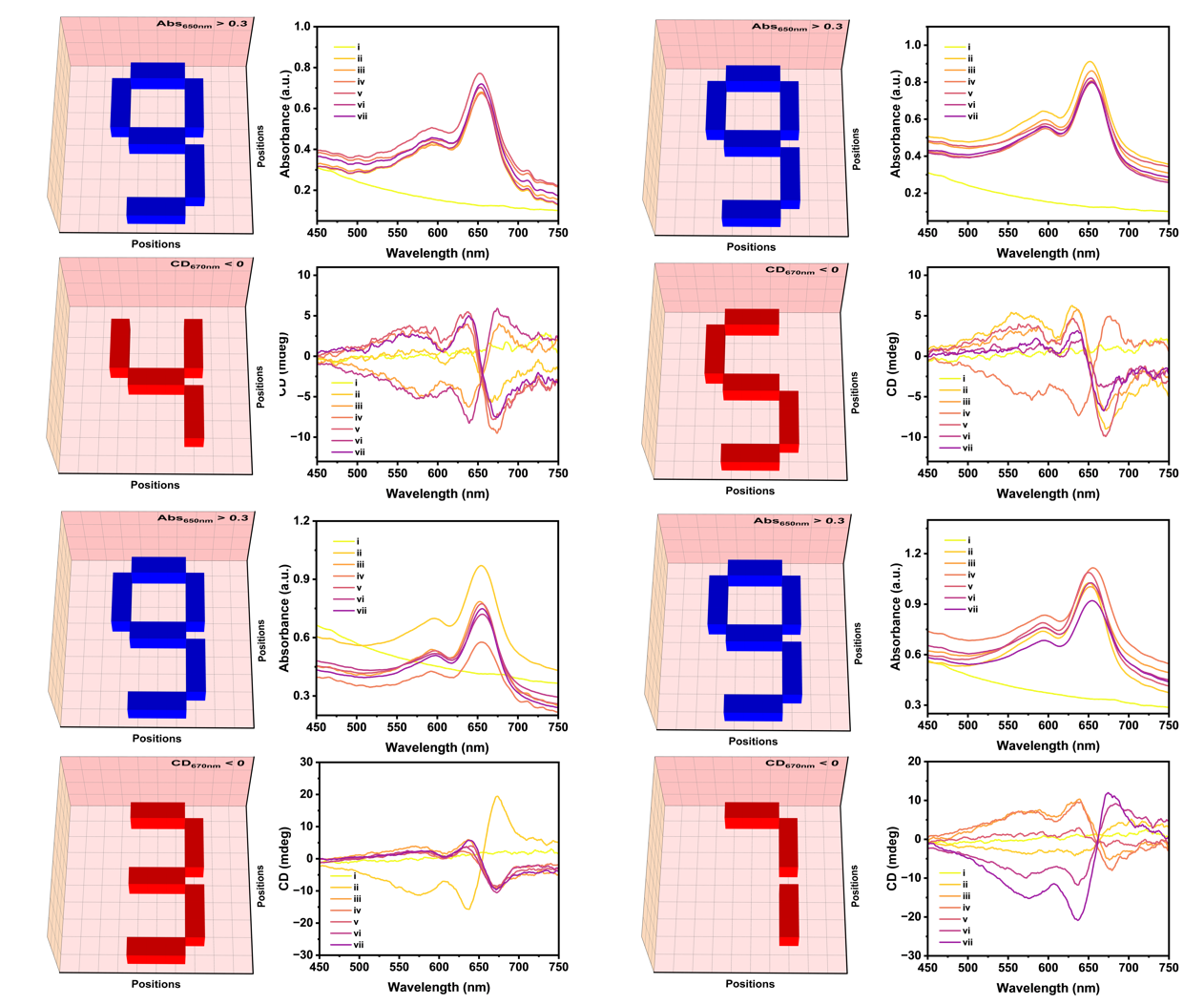


**Figure S13.** UV-Vis and CD spectra for PDA patterns at different positions, in the four samples shown in Fig. 4 in the manuscript.
